# Follicular Fluid and Blood Monitorization of Infertility Biomarkers in Women with Endometriosis

**DOI:** 10.3390/ijms25137177

**Published:** 2024-06-29

**Authors:** Ana Teresa Brinca, Ana Maria Peiró, Pilar Matallín Evangelio, Irene Eleno, Antonio Helio Oliani, Vladimiro Silva, Luís F. Vicente, Ana Cristina Ramalhinho, Eugenia Gallardo

**Affiliations:** 1 Health Sciences Research Centre, Faculty of Health Sciences, University of Beira Interior, 6200-506 Covilhã, Portugal; anabrinca99@gmail.com; 2 Pharmacogenetic Unit, Clinical Pharmacology Department, Alicante Institute for Health and Biomedical Research (ISABIAL), Dr. Balmis General University Hospital, 03010 Alicante, Spain; peiro_ana@gva.es; 3 Institute of Bioengineering, Miguel Hernández University, 03202 Elche, Spain; 4 Unidad de Reproduccion, Hospital General Universitario de Alicante, 03010 Alicante, Spain; pmatallin@gmail.com; 5 Unidad de Reproduccion, Servicio de Ginecologia y Obstetricia, Hospital General Universitario Dr. Balmis, 03010 Alicante, Spain; eleno_ire@gva.es; 6 Assisted Reproduction Laboratory, Cova da Beira Local Health Unit, 6200-251 Covilhã, Portugal; aholiani@gmail.com; 7 São José do Rio Preto School of Medicine, Gynaecology and Obstetrics, São José do Rio Preto 15090-000, Brazil; 8 Ferticentro—Centro de Estudos de Fertilidade S.A., 3000-316 Coimbra, Portugal; vladsilva@ferticentro.pt; 9 Procriar—Centro de Procriação Medicamente Assistida, 4100-130 Porto, Portugal; 10 Centro PMA Hospital Lusíadas, 1500-458 Lisboa, Portugal; luisferreiravicente@gmail.com; 11 Laboratório de Fármaco-Toxicologia, UBIMedical, University of Beira Interior, 6200-284 Covilhã, Portugal

**Keywords:** endometriosis, infertility, biomarker, follicular fluid, serum, plasma

## Abstract

Infertility is recognized globally as a social disease and a growing medical condition, posing a significant challenge to modern reproductive health. Endometriosis, the third-most frequent gynecologic disorder, is one of the most common and intricate conditions that can lead to female infertility. Despite extensive research, the etiology, malignant transformation, and biological therapy of endometriosis remain unknown. Blood and follicular fluid are two matrices that have been carefully studied and can provide insights into women’s health. These matrices are clinically significant because they contain metabolites closely associated with women’s illness stage and reproductive outcomes. Nowadays, the application of metabolomic analysis in biological matrices may be able to predict the outcome of assisted reproductive technologies with greater precision. From a molecular viewpoint on reproductive health, we evaluate and compare the utilization of human follicular fluid and blood as matrices in analysis for diagnostic and assisted reproductive technology (ART) predictors of success for endometriosis patients. In the follicular fluid (FF), plasma, and serum of endometriosis-affected women, researchers identified dysregulations of oxidative stress, upregulation of several immune factors, and aberrations in energy metabolic pathways. The altered signatures negatively correlate with the overall oocyte and embryo quality and fertilization rate.

## 1. Introduction

Endometriosis is an enigmatic disease and a debilitating gynecologic condition recognized as an individual, medical, and social concern due to its prevalence in women of reproductive age worldwide [1,2,3,4]. Even though this clinical condition is one of the leading causes of female infertility [2,3], its origin [3], etiology, pathogenesis [3,5,6], malignant transformation, and laboratory management are yet not well understood [3], with very few non-invasive diagnostic markers or curative treatments in sight [1].

The conventional description of endometriosis relies on the presence of endometrial, stromal, and glandular tissue in abnormal sites outside the uterus [1,4]. Nevertheless, there is a great deal of variation among individuals regarding the disease’s phenotypic manifestations and the severity of any accompanying symptoms [1], all of which significantly decrease the quality of life [4]. This disease has several phenotypes, including superficial endometriosis, deep infiltrating endometriosis, and ovarian endometrioma [7,8]. However, it is usually categorized according to 4 stages of clinical severity, with Stage I being the least severe and IV the most severe phase [4].

Even while endometriosis is connected to several clinical abnormalities, such as persistent inflammation [1], chronic pelvic pain [1,3,7,9], dysmenorrhea, adnexal masses, and abnormally high estrogen concentrations in the endometrial-like tissue that grows outside the uterus, its exact origin is still mostly unknown [1,4]. This might be explained by the clinical condition’s complicated and multifaceted character, to which genetic, endocrine, environmental, and immunological components have all been previously linked [1].

**Figure 1:**
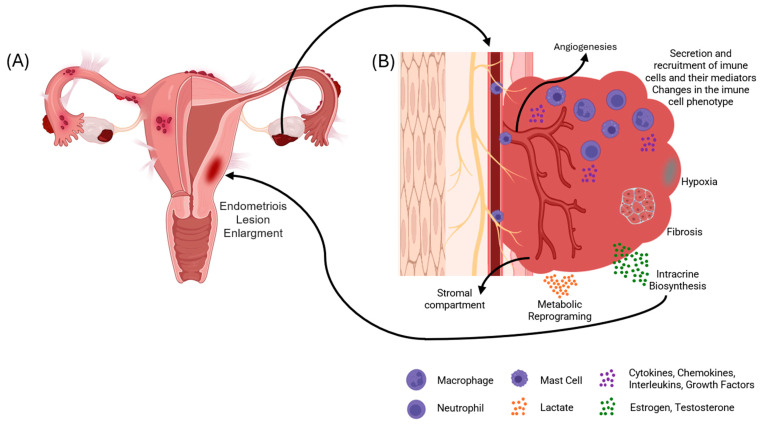
Schematic representation of the endometriotic lesion. (**A**) illustrates a female reproductive system with endometriosis. (**B**) shows the behavior and development of the endometriosis tissue.

Nowadays, it is known that oxidative stress, genetic mutations, inflammation, cell invasion, and angiogenesis have important roles in the etiology of endometriosis [2]. Figure 1 portrays a schematic representation of the endometriotic lesion. The disease’s negative impact can generate reproductive dysfunction and infertility [1], affecting oocyte quality and maturation, impairing embryonic development, lowering implantation competence, and reducing clinical pregnancy rates [2,7,10]. Lower fecundity in these women is attributed to anatomic changes, such as adhesions, that disrupt folliculogenesis and ovum pick-up mechanisms [11,12]. Women with endometriosis have impaired follicular development, which leads to altered expression patterns in the blood, serum, and follicular fluid (FF) [13].

The gold standard for endometriosis diagnosis encompasses invasive procedures, surgery, and a histopathological exam. All these procedures are highly invasive, time-consuming, and with a low efficacy rate, never completely managing the disorder. Although a greater knowledge of the pathophysiologic mechanisms and bigger datasets are required, non-invasive biomarkers, such as those found in the field of omics, may help in the diagnosis. Gaining a deeper comprehension of the molecular pathways underlying the pathogenesis of endometriosis through this information may help with the early non-invasive diagnosis of this pathology. It becomes, therefore, imperative to unravel new forms of detecting and managing this clinical condition to provide these women with a more disease-free life. Consequently, a deep comprehension of the pathophysiology of endometriosis is necessary for establishing innovative diagnostic and therapeutic strategies for this crippling condition. This review aims to address a wide range of endometriosis-related biomarkers expressed in three different biological matrices—FF, serum, and plasma—as well as their detection methods and subsequent fertility outcomes.

## 2. Metabolomics Biomarkers

**Figure 2:**
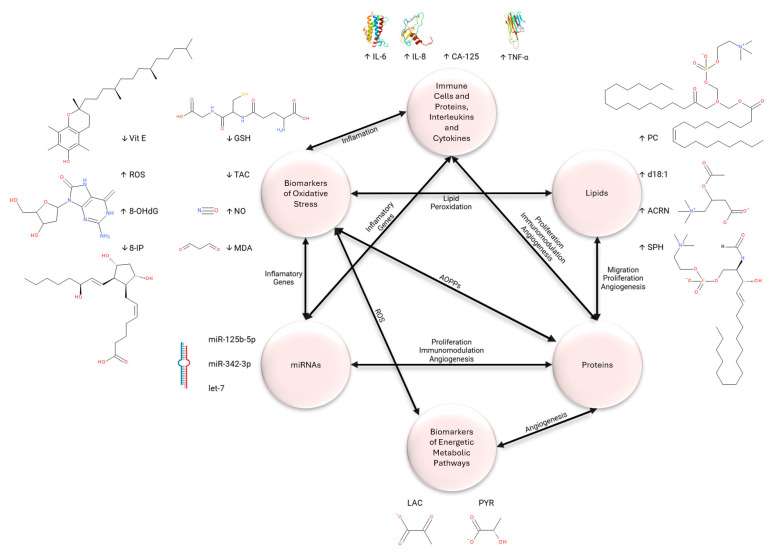
Schematic representation of the interactions between the different groups of compounds. Reference to the most cited endometriosis biomarkers found in the FF, serum, and plasma. (↑) Corresponds to high levels of the compound in question when compared to controls. (↓) Corresponds to low levels of the compound in question when compared to controls. Biomarkers of Oxidative Stress: (Vit E) vitamin E; (ROS) reactive oxygen species; (8-OHdG) 8-hydroxy-20-deoxyguanosine; (8-IP) 8-isoprostane; (GSH) glutathione; (TAC) total antioxidant capacity; (NO) nitric oxide; (MDA) malondialdehyde. Immune Cells and Proteins, Interleukins and Cytokines: (IL-6) interleukin 6; (IL-8) interleukin 8; (CA-125) cancer antigen 125; (TNF-α) tumor necrosis factor- α. Lipids: (PC) phosphatidylcholines; (d18:1) sphingolipid; (ACRN) acylcarnitines; (SPH) sphingomyelin. Biomarkers of Energetic Metabolic Pathways: (LAC) lactate; (PYR) pyruvate. (AOPPS) advanced oxidation protein products.

Conventional methods tend to be imprecise and ineffectual, presenting some analytical limits concerning the identification and analysis of biomarkers associated with oocyte development and the prediction of their quality and viability. Therefore, metabolomic analysis in biological matrices may be able to more accurately predict whether assisted reproductive technology (ART) will be successful [14]. The study of metabolomics has become an essential tool for investigating health, illness, clinical biomarkers, and treatment development [15]. Concentrating on the dynamic alterations of all small molecules in reaction to an organismal disturbance can offer a profound understanding of etiopathogenesis and the identification of distinct biomarkers for a range of metabolic disorders associated with the causes of health and illness [15,16]. Low-molecular-weight compounds derive from a range of cellular and biological activities. Consequently, they can establish the ideal correlation between gene expression and their corresponding phenotypes by offering vital information on genomic, epigenomic, matrix, and environmental results of a cell, tissue, or organism function. However, a metabolic inquiry may be more beneficial than the study of genomes, transcriptomics, and proteomics since metabolites may linked to certain biological activities and processes in systems, cells, or tissues [17,18,19,20,21]. These molecules can be accessed via biological matrixes like plasma, serum, and FF, being distinguished by their biological and medical signature. These enable the quantitative measurement of the dynamic chemical reactions in living systems in response to a pathophysiological insult or genetic variation [20,21]. Numerous clinical investigations have demonstrated that metabolomics may be employed, ushering in a new era of improved clinical diagnostics, early illness identification, therapy prediction, and monitoring of treatment efficiency [15,22]. Metabolomics can be utilized in reproductive medicine to identify and quantify low molecular weight metabolites essential for cellular activity in these matrixes [23]. The causes of infertility will then be linked to a metabolic imbalance [23,24]. These attributes render them appealing candidates for biomarkers, ideal for investigating human oocytes and embryos, as well as their growth [20,21]. Figure 2 is a schematic representation of the most cited endometriosis biomarkers found in the FF, serum, and plasma. The different groups of compounds are interconnected through several metabolites, reactions, and pathways, demonstrating the complexity of the analytes described, as well as the importance of their origin and function.

## 3. Follicular Fluid (FF)

FF is a liquid that fills the follicular antrum [25] and serves as a complex microenvironment mediator for germ cell–somatic cell communication [16]. It encompasses a variety of metabolites, such as hormones, proteins, anticoagulants, electrolytes, reactive oxygen species, antioxidants, and a diversity of cells from the immune system [25], enabling different reactions to take place that are crucial to oocyte growth [16]. It is produced by the diffusion of serum, transudate of plasma, and metabolites that are produced in the follicle wall and are subsequently modified by theca and granulosa cells [14,20,23,26]. Moreover, compounds resulting from ovarian cells’ biological activity and local follicular metabolic processes are also present since the oocyte matures and differentiates in vivo within this cellular matrix [14,20,23]. To guarantee proper oogenesis and ovarian folliculogenesis, regulatory extra- and intraovarian elements must always be kept in balance [24,25]. Alterations in the interplay of these variables may lead to aberrant folliculogenesis and oocytes of lower quality [25]. Disruption of the intrafollicular environment under various clinical circumstances may affect the likelihood of becoming pregnant if therapy and/or corrections are not performed promptly [26]. Therefore, a deeper comprehension of FF and its metabolic profile is essential for the investigation of several disorders, including endometriosis. Considering that, regarding ART, FF is a non-invasive matrix that collects biological information on fertility while reflecting changes in the patient’s milieu, it has become a vital source of knowledge. The molecular and biomolecular hallmark of FF has sparked a lot of attention, prompting numerous studies searching for new targets that allow for the evaluation of the oocyte’s development [14,23,27]. Consequently, a complete knowledge and characterization of FF may be helpful in identifying metabolites that might impede normal female function and promote infertility.

Table 1 contains the results mentioned below and further research regarding infertility biomarkers profiling in the FF of women with endometriosis, as well as the respective fertility outcomes.

### 3.1. Biomarkers of Oxidative Stress

Oxidative stress (OS) is defined as an imbalance between the generation of reactive oxygen species (ROS) and innate antioxidant systems [28,66]. OS affects a wide range of physiological and pathological aspects of the female reproductive system [66], and its connection with endometriosis has drawn particular attention in recent years [29]. As the disease progresses, inflammation increases and generates ROS, boosting the expression of inflammation genes. This will accelerate the development of endometriosis and other pathological processes that affect the female reproductive system [28,29,66]. When present in appropriate quantities, free radicals participate in several physiological processes, including folliculogenesis, oocyte maturation, quality, activation, and implantation, germ cell activity, embryonic development, hormone signaling, tubal function, ovarian steroidogenesis, cyclical endometrial alterations, and fetoplacental development [30,66]. Nonetheless, when ROS levels remain excessively high, they can cause significant damage to cell structures [66]. Therefore, the antioxidant status and OS markers in human FF surrounding oocytes may be related to the outcomes of IVF and embryo transfer [29,30,32], making it imperative to study this dynamic. Additionally, endometriosis-FF-related cases are more likely to show a decrease in fat-soluble antioxidants than in water-soluble antioxidants [33]. Several studies identified increased ROS in endometriosis patients. These correlated with poor oocyte and embryo quality, also decreasing the chances of women with endometriosis achieving pregnancy [31]. In contrast, Rajani and colleagues registered low levels of ROS in endometriosis patients. These were related to good spindle imaging results, with a higher number of grade 1 embryos, suggesting a possible role of endometrial receptivity accounting for lower pregnancy rates in these women [34]. Accordingly, patients who did not become pregnant presented lower ROS, suggesting that ROS occur at low concentrations in the FF and are perhaps even necessary for fertilization [35].

Endometriosis involves significant disarray in the production and metabolism of nitric oxide (NO) [36]. NO is a ubiquitous free radical [37], known as an intra and extra-cellular mediator [36], and largely produced by macrophages [67]. It contributes to a biological process of the ovary’s physiology and the microenvironment surrounding the ovum [36,37]. To facilitate meiotic maturation, fertilization, embryonic cleavage, and implantation, NO may postpone the aging of oocytes. Under some pathologic circumstances, decreased NO bioavailability may lead to anomalies in oocyte viability and developmental potential [36]. Regarding the levels of NO in endometriosis patients, these were found to be higher [31,37], with a tendency towards the polymorphism of allele GG. The existence of polymorphism GG leads to increased NO levels, causing a decrease in fertility derived from the degenerative oocyte [37]. NO presented a significant relationship with variables such as age, parity, dysmenorrhea, dyspareunia, state of endometriosis [37], and poor oocyte and embryo quality, also decreasing the chances of achieving pregnancy [31].

Proteins, lipids, and nucleic acids can be oxidized by ROS, altering cellular structure and function. The assessment of oxidative state is frequently performed using lipid peroxidation tests since lipids in lipoproteins and cellular membranes are important targets for peroxidation [68]. Advanced oxidation protein products (AOPPs) are novel markers of OS, crucial for inflammatory mediation in various chronic diseases. The AOPP concentration in the FF of endometriosis patients tends to be significantly higher [38]. FF lipid peroxidation levels in endometriosis women are also significantly higher [31,39,40], with these women later presenting a lower fertility rate [31,39]. A decrease in malondialdehyde (MDA) levels has also been described. This is a pro-oxidant marker negatively related to increasing female age [39,41]. Increasing age pertains to a decrease in the severity of the oxidative status but also a decreased chance of achieving pregnancy [39]. The study of MDA is an accurate parameter to measure ROS due to its quality as a good marker of the metabolic activity within the follicle [35]. Patients who became pregnant had higher levels of MDA and total antioxidant capacity (TAC) in the FF, which positively correlated with the pregnancy rate. The antioxidant defense system has many components [42]. According to recent investigations, TAC impacts the number of good-quality embryos [30,40]. These support the idea that OS highly contributes to the reproductive potential of IVF and ICSI patients [30,31]. Fabjan and the research team studied the TAC, and their findings suggest that the levels of these compounds were lower in the FFs of interest. Healthy women presented higher TAC concentrations and, therefore, a positive association with clinical pregnancy rates [41,42]. However, when progressed to clinical pregnancy, women with endometriosis presented even lower levels of TAC [30,40]. Resolvin D1 (RvD1), a lipid mediator [69], has also been detected in the presence of endometriosis [41]. This presence might be controversial since RvD1 inhibits endometrial lesions and decreases pro-inflammatory factors [70], being rendered as a promising therapeutic agent [69].

Women with endometriosis may have increased OS due to impaired antioxidant systems manifested in changes in the expression of protective enzymes, poor scavenging, and a decrease in antioxidant compounds like vitamin E [36]. Vitamin E can block the beginning of lipid peroxidation or inhibit its propagation [28]. Conversely, endometriosis-afflicted women had higher vitamin E levels in their FFs, which should translate into decreased OS rather than the opposite [28]. This discrepancy may derive from two factors, with the first being the different levels referring to the stages of endometriosis. The second one relies on the exogenous administration of vitamin E, either from an antioxidant-rich diet or as a supplement, since this kind of substance is commonly provided to individuals who have endometriosis [35]. The FF was additionally examined for vitamin C, determining that endometriosis patients had lower concentrations of this vitamin. Since vitamin C is a strong natural antioxidant, decreased quantities may arise from overconsumption to offset ROS. A further claim argues that excessive vitamin C would significantly lower ROS concentrations, and certain levels of ROS are required for appropriate oocyte maturation and embryonic development [35].

Important demonstrations were also conducted with other compounds related to OS. By neutralizing harmful peroxides, glutathione (GSH), the most prevalent thiol in all mammalian cells, serves as the primary antioxidant defense mechanism [32]. Total GSH activity is vastly used to evaluate the endogenous antioxidant defenses [71]. GSH was lower in both patients with endometriosis [31,32,43] and in patients who had a low fertilization rate after ICSI [32] since the GSH levels positively correlated with the number of high-quality embryos [43]. As an oxidized derivative of deoxyguanosine, 8-hydroxy-20-deoxyguanosine (8-OHdG) is one of the most prevalent oxidative changes in mutagenic damage [42]. Therefore, it plays an important role as a biomarker of oxidation, particularly DNA damage, in granulosa cells and FF [42,72,73]. Additionally, it is negatively associated with the quality of oocytes and embryos in IVF and ICSI patients [72,73]. Research on follicular OS and systemic oxidative stress has shown that endometriosis-affected women have greater 8-OHdG concentrations in the FF [28,29,30]. Additionally, it generated a negative impact on the number of good-quality embryos [30], related to the low rate of good-quality blastocysts [32], further intertwining the overall pathogenesis of endometriosis to infertility [28]. As a class of prostaglandin F2-like molecules, 8-Isoprostane (8-IP) is a highly sensitive, chemically stable, and quantitative marker of OS. It derives from the peroxidation of phospholipid-bound arachidonic acid, catalyzed by free radicals [42,74]. F2-isoprostanes are considered the best available biomarkers of oxidative stress status and lipid peroxidation. The 8-IP levels were found to be lower in the FF of women with endometriosis [42].

### 3.2. Immune Cells and Proteins, Interleukins and Cytokines

Endometriosis is an aseptic inflammation that contributes to the decline of female fertility [44]. There is increasing evidence that autoimmune phenomena, including autoantibody production, may affect fertility, particularly in women with endometriosis [45]. Ectopic lesions, common in endometriosis, exhibit elevated expression of genes linked to immune cell recruitment, cytokine-cytokine receptor interactions, cellular adhesion, and apoptosis [1]. Furthermore, by producing and secreting immunosuppressive substances, accumulating various immune cell types, and expressing specific antigens, the aberrant endometria may defend itself against immune system destruction [3,5]. Women with endometriosis have lower clearance of endometrial debris in the peritoneal cavity due to increased regulatory T cell induction, NK cell dysfunction, decreased macrophage phagocytosis, and overexpression of cytokines, growth factors, and adhesion molecules. Changes in the immune response found in the peritoneal cavity and fluid can also be seen in FF through various immunologic components and markers [11,75]. These irregularities may influence the severity of endometriosis and result in different clinical behaviors [3,76,77]. Because the complex regulatory network between FF’s cytokines and active immune cells affects the quality and development of oocytes, potentially impacting ART, many research teams have focused on the chronic inflammation triggered by endometriosis [44]. The overactive inflammatory environment may interfere with normal folliculogenesis and oocyte maturation, leading to poor fertility outcomes. As a result of endometriosis lesions in the ovary or peritoneal cavity, the immunological profiles in the FF of endometriosis patients may also suggest immunological alterations in the systemic circulation or local inflammation [11,78]. Women with endometriosis often exhibit elevated levels of immune cells and cytokines that guide migration in their FF, indicative of an inflammatory response within the ovarian environment. This inflammation can impair endometrial receptivity, possibly due to progesterone resistance and altered endometrial gene expression, which also affect the oocyte-granulosa cell complex and ultimately modify the immune content balance [11,41,79]. Some studies have found the presence of autoantibodies and immune complexes in the FF, suggesting an autoimmune component to the disease. These autoantibodies may target endometrial and ovarian tissues, contributing to inflammation and tissue damage [44,59,80].

Cytokines are small, soluble signaling proteins, best known for their immunoregulatory properties but increasingly recognized as growth factors governing cell proliferation, differentiation, and function [44]. Interleukins are a subgroup of cytokines that present an abnormal profile in endometriosis [43,46,47,48,49,81,82,83,84,85]. Many studies have highlighted an overproduction of IL-6 [43,46,47,48,49]. High levels of this interleukin might correlate with risk factors such as an irregular menstrual cycle, dyspareunia, and dysmenorrhea [46]. IL-6 exhibits pro-adhesive effects and participates in angiogenesis, which may promote the development of ectopic lesions [81]. The excessive expression of corticotropin-releasing hormone (CRH) may boost the activation of inflammatory pathways linked to IL-6. The increase in these two compounds might compromise the intrafollicular microenvironment, affecting the qualities of the oocyte [48]. All these aspects could be associated with the pathogenesis of this painful disease [46], as well as a tendency towards endometriosis severity [47]. High concentrations of IL-8 were also detected [43,48,49]. These results correlated with a significantly lower percentage of mature oocytes and good-quality embryos [49]. IL-8 plays an active role in the development of ectopic lesions due to its angiogenic characteristics [81]. It induces endometrial cell attachment, proliferation, and neovascularization, which may improve the cells’ capacity for ectopically surviving [49,81]. It is proposed that its rise is also positively correlated with the overexpression of CRH [48]. However, some studies could not find a negative correlation between IL-8 and fertility outcomes, suggesting that the increase was possibly due to local production in the ovaries [44]. Numerous studies have shown that high IL-12 levels are negatively correlated with folliculogenesis, oocyte quality, and implantation [83,84,85], while others have stated that they are positively correlated with a low percentage of mature oocytes and good-quality embryos [49]. Moreover, severe endometriosis is related to higher IL-12 levels in other fluids [82]. Tumor necrosis factor- α (TNF-α) is another mediator that might be overexpressed due to CRH [48]. It regulates proliferation, immunomodulation, and angiogenesis, presenting cytotoxic and proinflammatory properties. In endometrial epithelial cells, both its mRNA and the protein itself are overexpressed during the proliferative phase. Later, during the early secretory phase, they fall, and then during the late secretory phase, when they occur in both epithelial and stromal cells, they rise again. Moreover, TNF-α induces the production of several growth factors [86]. Several studies found high concentrations of TNF-α in the FF of endometriotic patients [43,48,49]. These levels were negatively correlated with the cumulative embryo score per embryo [43]. As referred before, endometriotic stromal cells produce several inflammatory mediators, among them monocyte chemoattractant protein-1 (MCP-1) [87]. MCP-1 is a well-researched chemokine and a member of a small inducible gene family that recruits monocytes to injury and inflammation sites [50,88,89,90]. It is a member of a small inducible gene family that plays a role in the recruitment of monocytes and macrophages to injury and inflammation sites [3,81,89]. It controls their migration and infiltration, which are enhanced in the peritoneal cavity in endometriosis [91]. Furthermore, MCP-1 has pro-adhesive properties that might foster the growth of ectopic lesions and endometrial cell proliferation, improving the lifespan of ectopic endometrial cells [81]. MCP-1 levels were elevated in other fluids in women with endometriosis [3,89], relating to the severity of the disease [3,89]. Recent studies identified elevated levels of MCP-1 in the FF of women with endometriosis [90]. However, Han and collaborators did not find relevant differences between controls and cases [44], and some studies even found lower levels of the compound [11,92]. Additionally, several biomarkers, such as IL-1β, IL-23, resistin, aLN-l, IL-3, and IL-5, were also analyzed and related to fertilization outcomes, albeit to a smaller extent. IL-1β [47,49], IL-23 [51], and resistin [51,52] were highly present in the patients’ FFs, and their increase showed a tendency toward endometriosis severity. IL-1β is an inflammatory mediator [86] that contributes to NO generation in the human pre-ovulation follicles [37]. IL-23 is an inflammatory cytokine that participates in autoimmune diseases by promoting inflammation and may cause embryo implantation failure [51]. Adipose tissue, monocytes, and macrophages release resistin, which is linked to insulin resistance and obesity. Research has shown that resistin interacts with pro-inflammatory cytokines, leading to a significant impact on inflammation [52]. Additionally, FF anti-laminin-l antibody (aLN-l) showed an inverse correlation with metaphase II oocyte counts. The results highlight aLN-l presence in women with endometriosis, which may affect oocyte maturation, leading to reduced fertility [45]. Resistin levels were also correlated with endometriosis, showing a tendency towards endometriosis severity [51,52]. The remaining two interleukins had a smaller representation. Low levels of IL-3 might correlated with risk factors, such as an irregular menstrual cycle, dyspareunia, and dysmenorrhea, associated with the pathogenesis of this painful disease [46]. Along the same line, a lack of IL-5 also interfered with the menstrual cycle, dyspareunia, and dysmenorrhea in endometriosis [46]. IL-5 is a hematopoietic growth factor with anti-inflammatory properties that negatively correlates with endometriosis [90].

Regarding the immunologic proteins that compose the FF, Cao and team analyzed up-regulated metabolites such as IGLV7–46, IGHG2, GDN, and ITIH3 [57]. GDN, a heparin-binding protein, was identified as a potential protein biomarker. It is a serine protease inhibitor that inhibits urokinase, trypsin, and thrombin. Blocking thrombin promotes neurite extension [57,93]. According to the “retrograde menstruation” theory, GDN plays a part in astrocyte development and cell migration, which may connect to the endometrium’s distant metastases [93,94]. Moreover, GDN functions as an inhibitor of plasminogen activator, essential for the breakdown of basal membranes and extracellular matrix elements during ovulation and embryo implantation. The upregulation of GDN in the FF of endometriosis-associated infertility patients also reduces the production of plasmin, which may lead to female infertility [57]. The same group also discovered considerable downregulation of fetuin-B (FETUB), angiotensinogen (AGT), and corticosteroid-binding globulin (CBG). Additionally, AGT was discovered to be a putative protein biomarker connected to reproductive processes. AGT is a powerful modulator of blood pressure, body fluid, and electrolyte balance and a crucial part of the renin-angiotensin system. AGT is linked to the development of pain associated with endometriosis and has a role in the ovulation process by controlling the production of progesterone [57].

Lastly, Mu-Tian Han and co-workers found evidence of significantly lower fertilization and cumulative live birth rates in women with endometriosis. The ratio of CD4+/CD8+ T cells in the FF was lower, even though the levels of IP-10, RANTES, and G-CSF were statistically greater, particularly in more severe scenarios. The rates of blastocyst development and fertilization were adversely correlated with the concentrations of IP-10. IP-10 inhibits CYP19A1 receptor and FSH synthesis in human granulosa cells. These have a strong relationship with IVF success. Furthermore, CD4+ and CD8+ cells have increased levels of its receptor (CXCR3) [44]. IP-10 belongs to the CXC subfamily and is associated with autoimmune diseases [95]. Proinflammatory stimuli from different cells may lead to its production [81]. Consistent results in various studies have correlated peritoneal fluid in endometriosis patients, ectopic lesions, and RANTES in FF. To participate in the inflammatory response, RANTES can gather inflammatory cells from a nearby lesion. Proinflammatory cytokines and angiogenic factors will be produced, favorably affecting the occurrence and progression of endometriosis [95]. G-CSF promotes the growth and development of neutrophil precursors into fully formed neutrophils. It can also stimulate the mobilization of bone marrow-derived hematopoietic stem cells. Another marker for endometriosis is related to the elevated levels of G-CSF [90,96]. These findings imply that changes in the cytokines and lymphocyte subsets in endometriosis-affected women may impact oocyte growth, leading to less successful ART [44].

### 3.3. Lipids

The specific goal of lipidomics, a subset of metabolomics, is to profile hydrophobic molecular species in various matrixes [97]. Lipid pattern changes are frequent in several disorders and can be driven by diseases, exposure to toxins, genetic changes, or the environment [53]. Lipidomic profiles in FF are not widely replicated, with research constrained by small sample sizes. However, given that FF in ART is very accessible, a number of indicators seem attractive targets for additional diagnostic investigation [97]. A wide variety of lipids, including glycerophospholipids, fatty acids, carnitines, monoacylglycerols, lysophosphatidic acids, lysophosphatidylglycerols, diacylglycerols, lysophosphatidylcholines, phosphatidylserine, lysophosphatidylinositols, and phosphatidic acid, exhibit potential biomarker characteristics [2,54,97]. Analysis shows that lipids are represented differently depending on their pathways, and these play a significant role in the progression of endometriosis [53]. Marianna and coworkers found evidence that the FF of endometriosis patients presented high concentrations of overall phospholipids [2], while some studies documented a low presence of unsaturated lipids [55]. In the FF of endometriosis women, there is a deficiency of phospholipids associated with cellular activities such as transcription, signal transduction, enzyme regulation, secondary messengers, and transport. These can, therefore, be related to embryo quality and fetal development since they show an insufficient response to the activation of ultimate follicular maturation. Furthermore, minor concentrations of phosphatidylcholine, phosphatidylserine, phosphatidylglycerol phosphate, and phosphatidylinositol bisphosphate were also found [53]. On the other hand, phosphatidylcholines (ChoGpl) and sphingolipids are often more prevalent. These two vital lipid types have an intricate relationship with the inhibition of apoptosis. The most significant phospholipid subclass, ChoGpl, is involved in cell signaling, membrane shape, and proliferation, all of which can lead to the development of malignant tumors. The phospholipase A2 (PA2) enzyme, overexpressed in endometriotic lesions, uses the rise in ChoGpl as a substrate. Furthermore, lysophosphatidic acid, a lipid implicated in endometriosis, cancer, and cell proliferation, is produced by PA2 [98]. Sphingolipids are bioactive chemicals that play many roles by regulating essential cellular operations like cell division, differentiation, signal transduction, cell recognition, and death [53,56,99]. Several biological diseases, including cancer, have been linked to modifications in the metabolism of these lipids [53,56]. Differential metabolite lysophosphatidylcholine (LysoPC) plays a role in angiogenesis, inflammatory reactions, energy consumption, cell proliferation, and apoptosis. It can increase fertility by enabling spermatozoa to undergo an acrosome response. Particularly, LysoPC (18:0) and LysoPC (18:2(9Z,12Z)) were elevated [56].

### 3.4. Proteins

Several proteins identified in the FF may impact oocyte competence acquisition, maturation, and follicle development. Data from proteome studies that analyze the FF profile concerning endometriosis are becoming more abundant. The proteins under investigation have connections to lipid metabolism and transport, wound healing, complement and coagulation cascades, and cytoskeleton organization [100].

High-density lipoprotein (HDL) apolipoprotein E (ApoE) has anti-inflammatory, anti-atherogenic, and antioxidant effects. The multidirectional biological activity of this lipoprotein relates to its expression fluctuations during various gynecological diseases and disorders affecting female fertility. ApoE was not frequently associated with endometriosis, although some research tried to establish a connection between the two [101]. Liu and the research team discovered that in older women, lower retrieved mature oocytes may be linked to higher ApoE concentrations. Furthermore, there might be a connection between ApoE and spontaneous miscarriage, as well as notable variations in blastocyst quantity and quality. Moreover, ApoE4 carriers (ρ3/ϵ4, ϵ4/ϵ4) differed significantly, and the ApoE-ϵ4 allele was found to be substantially associated with endometriosis [58].

Chen et al. report that the FF of endometriosis-presenting women can quicken granulose cell apoptosis by controlling the expression of five apoptosis-related proteins: CASP3, BCL2, CASP9, BAX, and TP53. BCL2 expression was downregulated, whereas that of BAX, CASP3, CASP9, and TP53 was elevated. The apoptosis-inducing factors TP53, CASP3, and CASP9, as well as the survival factor BCL2, are involved in the proliferation and apoptosis of GCs. Furthermore, a total of five signaling pathways (cytokine-cytokine receptor interaction, apoptosis, modulation of actin cytoskeleton, MAPK, and p53 signaling pathway) and five protein biomarkers (INS, CXCL10, ICAM1, WIF1, and TNFRSF13C) were examined and linked to the clinical condition [59]. Marianna and the group also emphasized CXCL10’s involvement [2]. Unfortunately, these complicated systems have not yet been completely figured out to properly capture and incorporate the intricacy of endometriosis.

Through two research investigations, Turco and coworkers examined the various protein profiles in endometriotic women. In addition, they examined the women who succeeded in becoming pregnant and the ones who failed to do so [60,61]. In the first study, they found 62 proteins differentially expressed related to binding, immune response, cell division, cellular metabolism, and general function [60]. In the second one, they narrowed down their research to endometriosis women. The group that succeeded in becoming pregnant had some functional protein enrichment associated with stress response, suggesting a robust defense against wounding, oxidative stress, elevated catalytic activity, particularly kinase activity, induction of programmed cell death, a sign of apoptosis, and anti-apoptosis role. Functionalities associated with sensitivity to ROS and NO, as well as positive regulation of apoptosis, were prevalent in the group that was unable to conceive. Protease, endopeptidase, carboxypeptidase, and general hydrolase activity were among the processes with the highest representation of catalytic activity. The inflammatory response was positively regulated, as evidenced by the increased reactivity to stimuli, with a focus on the processes of leukocyte, lymphocyte, and B-cell activation. These findings imply that endometriosis causes variations in the expression of proteins in the follicular fluid, which may impact the success of conception. Apolipoprotein-AIV, transthyretin, complement factor-I, vitronectin, kininogen-1, and FAK1 were among the proteins that were identified as being implicated in endometriosis damage [61].

Regiani and team detected 37 proteins included in interaction networks. They highlighted elevated concentrations of kallikrein B and prothrombin, both related to coagulation processes, peroxiredoxin-2 and ferritin, part of the hemoglobin complex, and lastly, sex hormone-binding globulin, belonging to sterol metabolism [62]. There has been a new proposal suggesting that endometriosis may cause inflammation and hypercoagulability in women. Although coagulation and inflammation are distinct processes, it is becoming clearer that they are interdependent. In fact, the pathway of coagulation activation in endometriotic women is consistent with cyclic bleeding in endometriotic lesions, which leads to repetitive tissue damage and repair and, ultimately, platelet activation and aggregation [102]. The most common proteins identified involved the transport, binding, regulation, and metabolism of steroid activities, particularly sex hormone-binding globulin, according to an analysis of the key functions in the FF endometriosis group. These proteins may be connected to the steroidogenic cascade that maintains endometriosis or to normal processes involved in hormone generation and transport in the follicle [62].

### 3.5. Energetic Metabolic Pathways

The biochemical quality of FF has a significant impact on the success of ART and the subsequent development of the embryo up to the birth of healthy children [7,13,33,55]. Determining the low-molecular-weight chemicals in the FF is vital to highlighting the quality of this biological fluid. Due to their diverse metabolic roles, this group of substances comprises compounds that are either directly or indirectly involved in catabolic reactions associated with energy production [33]. Women with endometriosis have significantly lower levels of acetate, β-hydroxybutyrate, citrate [55], ascorbate [33], lysine, choline, aspartate, alanine, proline, leucine [2], homocysteine [50], and valine [2,55] in their FF. Reduced valine levels in this clinical condition correlate with inflammatory processes [55]. Nonetheless, the study led by Karaer and colleagues revealed statistically significant higher levels of the marker [13]. Regarding fertility outcomes, acetate and the quantity of recovered oocytes had a negative correlation [55]. The blood and FF homocysteine levels were positively correlated in the endometriosis group. A byproduct of the methionine cycle, homocysteine comprehends several disorders related to obstetrics, gynecology, and embryology. Numerous disorders are connected to elevated homocysteine levels, particularly autoimmune and inflammatory diseases. Elevations of homocysteine may produce free radicals, which could lead to an imbalance between antioxidants and free radicals. The pathophysiology of preeclampsia disease also involves homocysteine. High homocysteine levels are linked to an increased risk of early pregnancy miscarriages. Furthermore, homocysteine is involved in several pathways that are critical to the gametogenesis process [50].

Glucose, lactate, and pyruvate are interconnected molecules that ensure efficient energy utilization. Lactate is one of the primary products of the granulosa cells, and pyruvate cannot be transformed into it when it is delivered to the oocyte [74]. The evaluation of glucose gave rise to contradictory results. Some research groups detected high levels of glucose [13,55], positively correlating this increase with the number of monitored follicles [55]. On the other hand, low concentrations of this compound have also been described [2,7,33], highlighting activation of the anaerobic glycolysis pathway and mitochondrial dysregulation in endometriosis phenotypes. Overall, higher levels of lactate [2,7,13,33,55] and pyruvate [7,13] were described. In endometriosis, an increase in lactate is associated with inflammatory processes [7,55]. Lactate is a final product of anaerobic glycolysis, a vital metabolic fuel, energy source, and gluconeogenic precursor. Its production and accumulation belong to one of the four crucial steps involved in cell metabolism. Furthermore, lactate enhances migration, induces angiogenesis, and reduces mitochondrial energy production and ROS generation [103].

### 3.6. Other Compounds

Vitamin A (retinol) is a critical micronutrient required for various cellular functions, including stem cell control, differentiation, and metabolism. The pathobiology and pathophysiology of endometriosis may be affected by a reduction in vitamin A and its retinoic acid metabolites [104]. Biologically active retinoic acid (ATRA) is a metabolite of retinol that is necessary for many reproductive functions [63]. It is well-recognized that retinoic acid reduces inflammation. Progesterone controls the synthesis and function of retinoic acid in endometrial stromal cells. Retinoids are important in proper endometrial function and operate through many nuclear receptors [105]. Regarding their functions in ovarian folliculogenesis, oocyte maturation, and early embryogenesis, however, little is known [63]. Along with a reduction in the local generation of estradiol, ATRA significantly inhibits the growth of endometrial tissue cysts. The expression of retinoid receptors and the production of ATRA are modulated by shifting patterns of steroid exposure during the menstrual cycle. Corrective modulation of the endometrial synthesis of many components affected by endometriosis, including integrins, connexin-43, secretion, differentiation, and cytokines, is achieved by local ATRA [104]. The mean levels of ATRA are 50% lower in endometriosis-affected women. A reduced mean of high-quality grade I embryos and follicle size are associated with low amounts of ATRA. These results provide compelling evidence in supporting the hypothesis that ATRA is essential for oocyte development and quality and that endometriosis participants may have lower fecundity due to decreased ATRA synthesis [63].

## 4. Blood Specimens (Plasma and Serum)

The potential use of markers for the early detection of endometriosis has been extensively studied in the scientific literature over several years. However, while peritoneal markers are too sensitive to hormonal fluctuations and are correlated with the volume of peritoneal fluid, the analysis of serum and plasma markers has identified several intriguing molecules [106]. Blood-derived matrix biomarkers have specificity, sensitivity, and the capacity to link to disease activity so that disease progression may be monitored [15,106]. Even though they present themselves extracellularly, serum/plasma can be viewed as biofluids metabolically active. This interpretation is fundamental for their correct analysis and interpretation [15,22]. Being the second most used biofluid in metabolomics [22], blood serum, and plasma encompass a variety of applications linked to numerous illnesses [15]. These biological samples are dynamically regulated, and their compositions progressively change ex vivo [15] through their interactions with many tissues, offering an overview of metabolism that unifies multiple organs and systems, presenting a metabolic picture of global metabolism [22]. Many of these components are unstable and can be subject to oxidation, aggregation, or degradation [15]. Several studies have examined the qualitative and quantitative differences between serum and plasma, but only little differences have been discovered, rendering it challenging to choose between the two biofluids. The main difference between serum and plasma is clotting, and it has been demonstrated that substantial differences in the temperature and length of clotting can impact the metabolite composition of serum [15,22]. Identifying accurate biomarkers for endometriosis in different biological specimens may prevent the need for a laparoscopy, making the diagnosis process less intrusive and more accessible [107].

The findings presented above are included in Table 2, along with additional studies on the infertility biomarkers profiling in serum and plasma of endometriosis-affected women and the corresponding reproductive outcomes.

### 4.1. Biomarkers of Oxidative Stress

Several investigations compared the levels of oxidative stress indicators in the systemic circulation and follicular microenvironment of endometriosis-affected infertile women who underwent ART [28,29,35,108]. The excessive ROS generation in endometriosis might arise from increased glucose metabolism and defects in the mitochondrial respiratory system [108]. Women with endometriosis show higher serum concentrations of ROS, LPO, advanced oxidation protein products [108], and FOX1 [29]. On the other hand, they present lower TAC concentrations [28,29,108], superoxide dismutase (SOD) [35,108], catalase, and GSH [108]. These findings positively correlate with the concentrations of those in the FF, implicating both systemic and follicular oxidative stress in these women. However, Da Broi and the team found high levels of SOD and GSH [28]. Additionally, vitamin E plasma levels were significantly higher, which differed from the low levels presented on the FF [35]. These women generally have reduced antioxidant capacities and a changed pro-oxidant/antioxidant activity balance, which may influence folliculogenesis and proper embryo development.

### 4.2. Immune Cells and Proteins, Interleukins and Cytokines

Regarding the immunologic system, the research sought to determine whether cytokines and angiogenic molecules were essential follicular prognostic variables in predicting mature oocytes and high-quality embryos in endometriosis-affected women [45,49,51]. Significantly different serum and plasma concentrations of angiogenic molecules and cytokines have been identified in these women. Further investigation is necessary to ascertain the potential of these variables for oocyte and embryo developmental competence [49].

Cancer antigen 125 (CA-125) is currently the best clinical marker for endometriosis. Despite being found in different biological matrices, its sensitivity for diagnosing this clinical condition is low, particularly in the early stages [109,110,111,112,113,114,148]. Aside from endometriosis and ovarian tumors, elevated serum and plasma CA-125 levels have been linked to many malignancies, including non-gynecological disorders [109]. Nevertheless, women with endometriosis tend to have increased serum and plasma levels of CA-125 in the FF, with larger amounts associated with more severe phases [109,110,111,112,113,114,115,121]. It is important to emphasize that despite the importance of this compound, its study in FF has not yet been developed, and further investigation is required.

The majority of studies reported elevated levels of vascular endothelial growth factor (VEGF) and IL-12 in comparison to the levels examined in the FF [49,114,149]. Lee and colleagues found contradictory results, referring to low levels of VEGF, as well as IL-12 [120]. Angiogenesis and enhanced vascular permeability—two essential elements of the inflammatory response—are primarily stimulated by VEGF, a growth factor receptor binding [114,149]. When combined, these contribute to the development of endometriotic lesions [114] since the proliferation of endometrial cells in ectopic areas is largely dependent on the formation of new blood vessels. Its use as a biomarker has not been demonstrated, though. Moreover, earlier studies have shown increased VEGF levels in endometriosis patients, especially in those with more severe conditions [149]. IL-6 and glycodelin-A are vital regulators of proliferation, activation, motility, chemotaxis, adhesion, morphogenesis, and finally, implantation of various cells, including endometrial cells [126]. Both glycodelin-A [125,126] and IL-6 [49,113,115,126] concentrations were increased in the serum and plasma. In response to progesterone, endometrial epithelial cells release a glycoprotein A during the secretory phase. It also has a role in glandular morphogenesis and has an immunosuppressive impact, possibly through the inactivation of T and NK cells. As such, glycodelin-A has a contraceptive effect in the second portion of the secretory phase. It may also shield the embryonic semi-allograft against immunological assaults from the maternal immune system. Pathological disorders affecting the human endometrium, particularly endometriosis, exhibit aberrant expression of glycodelin-A [126]. IL-6 modulates the immune response by inhibiting cell growth in normal endometrial cells [113]. It is a multifunctional protein released in response to numerous signals, triggering an inflammatory response by stimulating B and T cell proliferation [126]. It also positively relates to oocyte maturation [49]. In endometriosis, however, there seems to be an increased resistance to this effect due to decreased receptors [113]. Furthermore, the endometrium of individuals with endometriosis produces more haptoglobin when IL-6 levels are elevated [126]. A key regulator of the development of cancer and chronic inflammation is IL-32. Previous research has demonstrated that patients with various inflammatory disorders have an increase in IL-32. This interleukin is a potent inducer of other proinflammatory cytokines, including TNF-α and IL-8. Given that endometriosis entails a persistent inflammatory state, a biological association with IL-32 seems reasonable. Moreover, the invasiveness and cellular viability of endometrial cells are markedly enhanced by IL-32 [121]. IL-8 is a mighty angiogenic agent and a significant chemokine that causes neutrophil chemotaxis. Processes such as adhesion, invasion, implantation, and proliferation of ectopic endometrial tissue occur in the peritoneal cavity of endometriosis-affected women. These processes are all mediated by IL-8, which further shields ectopic cells from apoptosis-related death. Thus, by directly stimulating the vicious cycle of endometrial cell attachment, which results in the transition from an acute to a chronic inflammatory stage, IL-8 may function as an autocrine growth factor in the endometrium and contribute to the pathophysiology of endometriosis. IL-8 levels correlate not only with disease severity but also with the quantity and size of active lesions. Previous records highlight elevated concentrations of this chemokine in the serum and plasma of endometriosis women [49,115]. Caccavo and team evaluated the aLN-l presence in the serum of women with endometriosis undergoing IVF and its impact on oocyte maturation and IVF outcome. aLN-l serum levels were significantly higher [45], and serum resistin levels matched the FF profile. Serum resistin and IL-23 levels were positively correlated with endometriosis stages III-IV. This was in line with the FF ratings. As described before, inflammation and immunological rejection in severe endometriosis may thereby lower the chances of embryo implantation, leading to infertility [51].

Using an exploratory approach of inflammation-related proteins, numerous investigations sought to understand the inflammatory profile in endometriosis. Whereas CXCL9 levels were down, AXIN1 and ST1A1 serum levels were up. However, only AXIN1 levels increased in plasma [91]. The cytoplasmic protein AXIN1 operates as a negative regulator of the Wnt signaling pathway by downregulating β-catenin. Elevated β-catenin expression occurs in endometriosis lesions, and there are clues that the Wnt signaling pathway may contribute to the etiology of endometriosis by facilitating cell migration and invasion [91,150,151,152]. ST1A1 catalyzes the sulfur conjugation of bile acids, medications, neurotransmitters, and hormones, in addition to being implicated in the metabolism of estrogens. Given its sensitivity to inflammatory activity, ST1A1 is considered a potential novel inflammatory biomarker. Furthermore, ST1A1 may signal elevated blood estrogen levels and the need for hormone treatment [91,153,154,155]. Lastly, the TPM protein family is essential to the cytoskeleton, playing a role in cellular contraction [110,156,157]. Numerous physiological processes, including adhesion, apoptosis, proliferation, motility of cells, receptor activity, and second messenger pathways, have been demonstrated to be significantly impacted by cytoskeletal proteins [110,158]. It is proposed that infertility may arise from an aberrant immunological process, which includes the generation of auto-antibodies [110,156,157], the development of autoimmune-related reproductive failures, and endometriosis-related infertility [110,159]. Tropomyosin 3 (TPM3), stomatin-like protein 2 (SLP2), and tropomodulin 3 (TMOD3) are the three endometrial antigens belonging to the TPM family. Significantly higher serum antibody levels were observed against the epitopes from the immunodominant region of the proteins TPM3, SLP2, and TMOD3. Anti-TPM3a, anti-TPM3c, anti-TPM3d, anti-SLP2a, anti-SLP2c, anti-TMOD3b, anti-TMOD3c, and anti-TMOD3d serum antibodies may represent novel indicators for the early detection of endometriosis [110]. Likewise, TPM3 could be involved in the early stages of reproduction and infertility brought on by endometriosis [110,156,157].

### 4.3. Lipids

The blood is composed of a variety of lipids. Abnormal lipid metabolism results in the production of free fatty acids, which are important chemical markers that affect cell development, differentiation, and metabolism by influencing gene expression. The fatty acid levels within the oocyte and their concentration in the surrounding environment might affect the developmental competence of the oocyte and the implantation of the embryo. It has been suggested that these aberrant metabolite levels cause many endoplasmic reticulum stress signals that are detrimental to the oocyte. Regarding this category, target analytes included acylcarnitines [53,117], glycerophospholipids [53,117,160], sphingolipids [53,117,119,160,161], phosphatidylcholines [53,117,119,161], sphingomyelins [53,117,119,161], phosphatidylethanolamines, di- and triglycerides [161], and glycerophosphatidylcholine [107]. The findings indicate that endometriosis aligns with higher concentrations of these substances, which may influence lipid-associated signaling pathways and inhibit apoptosis since they control several processes, such as migration, proliferation, and apoptosis [53,117,119]. The establishment and subsistence of ectopic lesions outside the endometrium suggest an altered cellular state for pathological hyperplasia. Furthermore, acylcarnitines in human plasma proved to be an accurate indicator of the occurrence of endometriosis. Acylcarnitines derive from carnitine and fatty acids. Their purpose is to transfer fatty acids into the mitochondria for beta-oxidation. One possible application of acylcarnitines in a diagnostic paradigm is to predict the existence and stage of endometriosis [118]. Thus, Dutta and the team reported low overall lipid concentrations [107].

### 4.4. Energetic Metabolic Pathways

Increased glucose metabolism was also analyzed in blood specimens. This resulted from abnormalities in the respiratory system of the mitochondria, which were most likely caused by endometriosis’s excessive production of ROS [108]. The serum of endometriosis women was rich in alanine, leucine [107,122], lysine, proline, phenylalanine [122], homocysteine, citrate [108], succinate [107,108], lactate, 3-hydroxybutyrate, valine, threonine, and 2-hydroxybutyrate [107]. Leucine, lysine, proline, and phenylalanine strongly correlated to advanced stages of this clinical condition [122]. The levels of homocysteine in the blood were positively correlated with those of FF [50], and the pyruvate metabolism was also upregulated [160]. Compounds such as glucose, isoleucine, and arginine were decreased [107]. The plasma metabolomic profile of endometriosis patients showed rising lipoproteins, fructose, choline-containing metabolites, valine, and lysine, arginine, whereas creatinine concentrations were down. The pathophysiologic events that occur throughout the disease are correlated with these metabolic alterations in the plasma metabolomic profile [123].

### 4.5. miRNA

Understanding medical disorders and diseases is also dependent on genetic information. Through the study of DNA, it is possible to identify genetic variants linked to illnesses or ailments that might impact treatment response, illness progression, or susceptibility to certain diseases [162,163]. Transcribed from DNA, RNA has a variety of functions in the expression of genes. It reflects gene expression levels that can indicate disease and serve as a biomarker [128,162,163,164]. Plasma samples from endometriosis patients encounter several types of miRNAs. Human miRNAs are non-coding, single-stranded, highly conserved RNAs with 21–25 nucleotides that bind to corresponding mRNAs to control translation and destruction, regulating 60% of genes. Evidence suggests that dysregulation of miRNA is linked to benign and malignant disorders with similar signaling pathways to endometriosis [128,164].

Dabi and colleagues sequenced several miRNAs, and their investigation determined that miR-124-3p and miRNA-548 were highly associated with endometriosis. miR-124-3p participates in the pathways of PI3K/Akt, mTOR, STAT3, NF-κB, ERK, FGF2-FGFR, MAPK, PLGF-ROS, and GSK3B/β-catenin, originating ectopic endometrial cell invasion and proliferation [128]. Several teams analyzed miR-125b-5p, an oncogene that may be involved in the invasion and etiology of cancer [130,133,135,136]. It has been linked to chemotherapeutic resistance and is upregulated in breast, bone, and lung cancer [135]. Additionally, miR-342-3p was also upregulated, being suggested as a circulating biomarker of endometriosis both in plasma [133,136] and serum [130,135].

To diagnose and manage endometriosis, Gu and colleagues looked at endocrine resistance pathways, nucleobase-containing chemical metabolic processes, cellular nitrogen compound biosynthesis activities, and heterocycle metabolic processes. They demonstrated that endometriosis is associated with the downregulation of hsa-let-7a-5p, hsa-let-7b-5p, hsa-let-7d-5p, and hsa-let-7f-5p [129]. Accordingly, Cho and the study team also discovered a downregulation of the let-7 family in the serum of patients with endometriosis. This family has a conserved sequence and function. Its dysregulation is associated with cell differentiation [129,140], tumor suppression [140], and cell-related diseases. They can target and bind to KRAS polymorphism linked to endometriosis pathogenesis [129]. Low levels of let-7b expression were also described in serum specimens [130].

Some miRNAs are targets of controversy. miR-320a was found upregulated in the serum [131] but downregulated in plasma samples [130]. Its carcinogenic properties rely on the PI3K/Akt pathway and STAT3 signal, which are involved in the pathogenesis of endometriosis [131]. In the same line, miR-22 was found to be upregulated in serum [131] but downregulated in plasma [136]. This miRNA targets the regulation of HIF-1 a, and its downregulation implicates several tumors [136]. While miR-451 was shown to be elevated in serum, it was downregulated in FF samples. miR-451 downregulation in oocytes impacts pre-implantation embryogenesis, inhibiting the Wnt signaling pathway [65]. Suryawanshi and collaborators detected overexpression of miR-196a and miR-196b [143], whereas Pateisky and the research team reported low expression of miR-196b [139]. Expression of miR-196b is consistently downregulated in ectopic sites of lesion implantation and depends on both the menstrual cycle and the medical condition. MiR-196b is an antagonist in the formation of ectopic lesions. Its overexpression in endometrial and endometriosis stroma cells inhibits cell proliferation and promotes apoptosis [139].

## 5. Conclusions

The vast majority of women affected by endometriosis exhibit a range of symptoms that negatively impact their mental, emotional, and physical health. The categorization of this clinical condition has remained controversial and complex due to its many manifestations. In the past, anatomy, histology, and disease load were the primary factors used to establish “surgical staging”; however, prognostic significance has recently been included. Furthermore, this condition hinders many couples from achieving fertility. In recent years, there has been a notable surge in research attempting to close existing knowledge gaps. Scientific and technological advancements have improved the prognosis for affected women, making ART more achievable. A comprehensive examination of FF, serum, and plasma enables the identification of several biomarkers from various pathways. Consequently, these biological matrices help develop novel indicators and provide fresh insights into endometriosis. Regarding OS indicators, ROS and NO have been found to be upregulated. The investigation has focused on the overflow of 8-OHdG. Several studies have recently concentrated on the immunologic components present in these matrices. For example, the upregulation of interleukins IL-6 and IL-8 has been frequently described. Alongside CA125, a well-known marker of endometriosis, these two interleukins could also serve as indicators. Imbalances in energetic metabolic pathways are common among these patients. TAC is downregulated, while glucose metabolism has shown contradictory results. Glucose concentrations did not yield consistent results; however, high lactate levels have been consistently described. Additionally, the altered metabolic signatures negatively correlate with overall oocyte and embryo quality, as well as the fertilization rate. Even though some metabolites showed a positive correlation in FF and blood specimens, many did not follow the same patterns, indicating that further studies are needed to improve the results and create accurate correlations. Overall, studies that analyze markers found in the FF are more prone to relate them to the fertility outcomes of endometriosis women. The compounds described in plasma and serum align with the clinical condition, but few results describe further infertility implications. These findings may offer an alternative for the diagnosis of endometriosis-related infertility and provide a perspective on how ART success rates could be improved. Further studies in various health-related disciplines are needed to fully understand this condition and all its complexities.

## Figures and Tables

**Table 1:** Infertility biomarkers profile in the FF of women with endometriosis. (↑) Corresponds to high levels of the compound in question in FF samples when compared to controls. (↓) Corresponds to low levels of the compound in question in FF samples when compared to controls.

Marker	Detection and Quantification	Oocyte and Embryo Quality, Fertilization Rate	Other Characteristics	Endometriosis Stage	Age (Years)	N° Patients/Controls	Ref.
↓ Fatty acids ↑ CH2NH2 phospholipids ↑ Lipids ↑ Lactate ↓ Leucine ↓ β-glucose ↓ α-glucose ↓ alanine ↓ Lysine ↓ Phosphocholine ↓ Choline ↓ Valine ↓ Aspartate ↓ Proline ↑ PTX3 ↑ CXCL8 ↑ CXCL10 ↑ CCL11 ↑ VEGF ↑ Insulin ↓ LDH	Immunoassay qPCR	↓ Retrieved oocytes ↓ MII oocytes	↑ insulin receptor mRNA levels in cumulus cells ↓ LDHB mRNA levels in cumulus cells	I/II III/IV	31–39	16/7	[2]
↑ 8-OHdG ↑ Vitamin E	HPLC Immunoassay	-	↑ oxidative damage to DNA in the follicular compartment	I/II/III/IV	<38	29/32	[28]
↑ 8-OHdG	Immunoassay	↓ Oocyte quality	-	I/II	30–36	19/32	[29]
↑ 8-OHdG	Immunoassay	↓ Good quality embryos	↑ TAC	II/IV	27–40	61/43	[30]
↑ NO ↑ ROS ↑ Malondialdehyde ↓ SOD ↓ GSH peroxidase ↓ GSH reductase ↓ Vitamin A ↓ Vitamin C ↓ Vitamin E ↓ Selenium ↓ Zinc ↓ Copper ↑ Iron ↑ Lead ↑ Cadmium	Reverse phase HPLC Immunoassay Analytical chemistry test Colorimetric assay Protein Estimation Kit Spectrophotometric	↓ Oocyte quality ↓ Embryo quality ↓ Total number of FF aspirated ↓ Oocytes retrieved ↓ Number of MII oocytes ↓ Fertilization rate ↓ Formation of grade I and II embryos	↓ TAC	III/IV	26–40	200/140	[31]
↓ Vitamin C (mg/L) ↑ 8-OHdG (ng/mL)	Colorimetric assay Immunoassay	↓ Fertilization rate after ICS ↓ Good quality blastocysts	↑ TAC (µmol/L) ↓ Total GSH (µmol/L)	-	31–40	35/64	[32]
↓ Glucose ↑ Lactate ↑ Ascorbate	Analytical chemistry test	↓ Retrieved Oocytes ↓ Mature Oocytes	↓ Fat-soluble antioxidants	-	23–35	145/35	[33]
↓ ROS	Immunoassay	↑ Mature MII oocytes	↑ Meiotic spindle present	-	29–37	56/63	[34]
↓ Vitamin C	Spectrophotometry	↓ Nº of follicles	↓ TAC	III/IV	-	23/68	[35]
↑ NO	Colorimetric assay	↓ Matured MII oocytes ↑ Oocyte aging ↑ GC apoptosis	↑ Protein nitration	-	35–38	10/18	[36]
↑ NO	PCR-RFLP	-	↑ Polymorphism of allele GG	I/II/III/IV	-	27/27	[37]
↑ AOPP	Spectrophotometric method described	↓ Blastocyst rate	↓ Progesterone	I/II/III	27–35	44/45	[38]
↑ MDA	Colorimetric assay	↓ Blastocyst rate	↑ Peroxidation levels were	III/IV	31–36	38/41	[39]
↑ LPO	Antioxidant capacity test Colorimetric assay	-	↓ TAC	-	<40	43/20	[40]
↑ MDA ↑ RvD1	HPLC Immunoassay	-	↓LH	-	35–42	22/29	[41]
↓ 8-IP	Immunoassay	-	↓ AMH ↓ TAC	-	31–35	72/48	[42]
↓ GSH ↑ TBP2 ↑ IL-6 ↑ IL-8 ↑ TNF-α ↑ GPX3 ↑ TRX	Immunoassay	↓ Total antral follicle count ↓ High-quality embryos ↓ Mature oocytes ↓ Cumulative embryo score per embryo	↑ Dose of gonadotropins ↓ Serum E2 on hCG day ↓ Serum AMH	-	31–38	31/34	[43]
↓ CD4+/CD8+ T ↑ CD45+/CD56+ NK ↑ CD45+CD14+ macrophages ↑ IP-10 ↑ RANTES ↑ G-CSF	Flow cytometry Immunoassay	↓ Antral follicle ↓ Blastocyst formation rate ↓ Ovarian reserve ↓ Retrieved oocytes ↓ D3 high-quality embryo rate ↓ Implantation rate ↓ Fertilization rate ↓ Clinical pregnancy rate ↓ Cumulative live birth rate of one oocyte retrieval cycle	↓ Serum AMH ↓ Serum E2 ↑ Inflammatory state	III/IV	26–32	40/40	[44]
↑ aLN-l	Immunoassay	↓ Metaphase II oocyte	-	-	26–43	35/50	[45]
↓ IL-3 ↓ IL-5 ↑ IL-6	Immunoassay	-	-	-	25–37	34/34	[46]
↑ IL-1β ↑ IL-6	Immunoassay	-	↓ Estradiol	II/III/IV	26–42	17/17	[47]
↑ Urocortin ↑ IL-6 ↑ IL-8 ↑ TNF-α ↑ RAGE	Immunoassay	-	↑ CRH	-	25–40	30/7	[48]
↑ IL-1β ↑ TNF-α ↑ IL-2 ↑ IL-4 ↑ IL-6 ↑ IL-8 ↑ IL-10 ↑ IL-12 ↑ INF-γ ↑ VEGF ↑ ADM ↑ Angiogenin	Immunoassay	↓ Oocyte maturity ↓ Embryo quality ↓ MII oocytes quality	-	III/IV	29–35	200/140	[49]
↑ Homocysteine	Immunoassay	-	-	-	30–38	29/29	[50]
↑ Resistin ↑ IL-23	Immunoassay	↓ Implantation rate ↓ Clinical pregnancy rate ↑ Abortion rate	-	I/II III/IV	26–36	76/40	[51]
↑ Resistin	Immunoassay	-	-	I/II III/IV	27–40	40/40	[52]
↓ Phosphatidylglycerol phosphate ↓ Phosphatidylcholine ↓ Phosphatidylserine ↓ Phosphatidylinositol bisphosphate ↑ Sphingolipids ↑ Phosphatidylcholines	ESI-MS	↓ Luteinization process ↓ Oocyte quality ↓ Embryonic cleavages	↓ Apoptosis regulation ↑ Cell proliferation ↑ Malignant tumors ↑ endometriotic lesions.	III/IV	26–35	10/10	[53]
↑ Fatty acids ↑ Carnitines ↑ Monoacylglycerols ↑ Lysophosphatidic acids ↑ Lysophosphatidylglycerols ↑ Diacylglycerols ↑ Lysophosphatidylcholines ↑ Phosphatidylserine ↑ Lysophosphatidylinositols ↑ Phosphatidic Acid	UPLC-MS	↓ Embryo quality ↓ Transferred embryos ↓ Implantation rates	-	-	33–39	18/22	[54]
↓ Acetate ↓ β-hydroxybutyrate ↓ Citrate ↓ Valine ↑ Glucose ↑ Lactate ↑ Unsaturated lipids	NMR	↓ MII oocytes recovered ↓ Fertilization rate ↓ Pregnancy rate	↑ Inflammatory processes	III/IV	35–42	8/10	[55]
↑ LysoPC(18:2(9Z,12Z)) ↑ LysoPC(18:0) ↓ Phytosphingosine	SWATHTM LC-MS	↓ MII rates ↓ Fertility rates	-	-	33–39	17/16	[56]
↑ IGLV7–46 ↑ IGHG2 ↑ GDN ↑ ITIH3 ↓ CBG ↓ AGT ↓ FETUB	LC-MS/MS LFQP PRM	↓ Oocyte development ↓ Oocyte quality ↓ Embryo implantation ↓ Endometrial receptivity	↑ Immune response ↑ Pelvic pain	I/II/III	28–35	20/10	[57]
↑ ApoE ↑ ApoE4	Immunoassay	↓ Retrieved mature oocytes ↓ Blastocysts and high-quality blastocysts ↑ Spontaneous pregnancy loss	↓ BMI	-	25–32	106/111	[58]
↑ BAX ↑ CASP3 ↑ CASP9 ↑ TP53 ↓ BCL2 ↑ TNFRSF13C ↑ BMPR2 ↑ FGF9 ↑ GPC3 ↑ SCYA1 ↑ ICAM1 ↑ IGFBP4 ↑ IGFBP6 ↑ IL-13RA2 ↑ CXCL10 ↑ MMP25 ↑ PDGFB ↑ CCL25 ↑ TGFBR1 ↑ TNFAIP6 ↑ EDA2R ↑ WIF1 ↓ IL23A ↓ XCL1 ↓ NAP1L4 ↓ HCRT ↓ WIF1	Immunoassay Real-Time PCR	↑ Granulosa cells’ apoptosis	-	I/II	<35	30/30	[59]
↓ PBX3 ↑ FAN ↑ IGLα ↑ IGLC1 ↑ Serotransferrin ↓ IL-2 ↑ CDCA2 ↑ TAK-1 ↑ PLGLA ↑ PPR3B	Protein Estimation Kit nanoUPLC-nanoESI-MSE	-	↓ Serum LH ↓ Regulation of apoptosis	III/IV	28–36	5/5	[60]
↑ Complement factor I ↑ Vitronectin ↓ VEGF ↑ Kininogen-1 ↑ FAK1 ↓ Apolipoprotein-AIV ↓ Transthyretin	2D SDSPAGE LC-ESI-MS/MS	-	↑ OX ↑ ROS ↑ Inflammatory response ↑ apoptosis	-	23–33	12/9	[61]
↑ Kallikrein B ↑ Prothrombin ↑ Sex hormone- -binding globulin	Mass spectrometry	-	↑ Coagulation	-	18–37	30/10	[62]
↓ Glucose ↓ Citrate ↓ Creatine ↓ Tyrosine ↓ Alanine ↑ Lactate ↑ Pyruvate ↑ Lipids ↑ Glycerol ↑ Acetoacetate ↑ 3-Hydroxybutyrate ↑ Acetone ↑ Threonine ↑ Glutamine ↑ Succinate	1H-NMR	-	↑ Lipolysis ↑ Beta -oxidation ↑ Anaerobic glycolysis pathway	-	30–41	50/29	[7]
↑ Lactate ↑ β-glucose ↑ Pyruvate ↑ Valine	NMR	-	-	-	28–39	12/12	[13]
↓ Retinol ↓ Retinoic acid	LC-MSMS HPLC-UV	↓ High-quality grade I embryos ↓ Follicle size	-	-	32–35	79	[63]
↑ Tetradecanal ↑ Octadecanal ↑ Hexadecanal ↑ Eicosamethyl-cyclodecasiloxane ↑ 4-methyl-2,4-bis(4-hydroxyphenyl)pent-1-ene	SPMS GC-MS	-	-	-	-	8/17	[64]
↓ miR-451	Immunoassay	↓ Quality MI oocytes ↓ Blastocyst-stage embryos	-	III /IV	-	30/184	[65]

**Table 2:** Biomarkers profile in the serum and plasma of women with endometriosis. (↑) Corresponds to high levels of the compound in question in blood and plasma samples when compared to controls. (↓) Corresponds to low levels of the compound in question in blood and plasma samples when compared to controls.

Marker	Detection and Quantification	Oocyte and Embryo Quality, Fertilization Rate	Other Characteristics	Endometriosis Stage	Age (Years)	N° Patients/ Controls	Ref.
↑ GSH ↑ SOD	Spectrophotometry	-	↓ TAC	I/II/III/IV	<38	43/44	[28]
↑ FOX1	Immunoassay	-	↓ TAC	I/II	30–36	27/44	[29]
↑ Vitamin E ↑ SOD	HPLC ELISA	↓ N° of follicles	↓ TAC	III/IV	-	23/68	[35]
↑ aLN-l	Immunoassay	↓ Metaphase II oocyte	-	-	26–43	35/50	[45]
↑ AXIN1 ↑ ST1A1 ↓ CXCL9	Immunoassay	-	-	-	29–44	94/28	[91]
↑ Citrate ↑ Succinate ↑ ROS ↓ SOD ↓ Catalase ↓ GSH	Colorimetric assay Immunoassay Biochemical assay Spectrophotometry NMR	-	↑ Glucose metabolism ↑ Lipid peroxidation ↑Advanced oxidation of protein products ↓ TAC ↓ Mitochondrial respiratory system	-	24–40	75/60	[108]
↑ CA-125 ↑ STX-5 ↑ LN-1	Immunoassay	-	-	I/II/III/IV	25–40	60/20	[109]
↑ Anti-TPM3a-autoAb ↑ Anti-TPM3c-autoAb ↑ Anti-TPM3d-autoAb ↑ Anti-SLP2a-autoAb ↑ Anti-SLP2c-autoAb ↑ Anti-TMOD3b-autoAb ↑ Anti-TMOD3c-autoAb ↑ Anti-TMOD3d-autoAb ↑ CA-125	Immunoassay MALDI-TOF/MS	-	-	I/II III/IV	25 -38	50/27	[110]
↑ CA-125 ↑ Leukocytes ↑ Fibrinoge	Immunoassay	-	-	-	25–35	48/38	[111]
↑ CA-125	Immunoassay	-	-	III/IV	26–42	42/46	[112]
↑ CA-125 ↑ IL-6	Immunoassay	-	-	I/II/III/IV	-	24/24	[113]
↑ Annexin V ↑ VEGF ↑ CA-125 ↑ sICAM-1	Immunoassay	-	-	I/II III/IV	24–44	232/121	[114]
↑ IL-6 ↑ IL-8 ↑ CA-125 ↑ hsCRP	Immunoassay	-	-	I/II III/IV	-	201/93	[115]
↓ PEDF	Immunoassay	-	↑ Pain related	-	25–37	43/28	[116]
↑ SMOH C16:1 ↑ PCaaC36:2/PCaeC34:2	ESI-MS/MS Biochemical assay	-	↑ Age ↑ BMI	III/IV	22–44	40/52	[117]
↑ Lauroylcarnitine ↑ Oleylcarnitine ↑ Myristoylcarnitine ↑ Hexadecenoylcarnitine ↑ Tetradecenoylcarnitine ↓ trimethylamine-N-oxide	UPLC-MS/ MS UPLC-ESI-Q-TOF	-	-	I/II/III/IV	25–39	25/19	[118]
↑ Glucosylceramide	LC-MS/ MS	-	↓ Apoptosis of shed endometrial cells	I/II/III/IV	22–44	38/24	[119]
↓ IL-12 ↓ IL-13 ↓ VEGF	LC-MS/MS	-	-	I/II III/IV	27–42	57/46	[120]
↑ CA-125 ↑ IL-32	Immunoassay	-	-	III/IV	33–36	50/35	[121]
↑ Resistin ↑ IL-23	Immunoassay	↓ Implantation rate ↓ Clinical pregnancy rate ↑ Abortion rate	-	I/II III/IV	26 -36	76/40	[51]
↑ IL-1β ↑ TNF-α ↑ IL-2 ↑ IL-4 ↑ IL-6 ↑ IL-8 ↑ IL-10 ↑ IL-12 ↑ INF-γ ↑ VEGF ↑ ADM ↑ Angiogenin	Immunoassay	↓ Oocyte maturity ↓ Embryo quality ↓ MII oocytes quality	-	III/IV	29–35	200/140	[49]
↓ Retinol ↓ Retinoic acid	LC-MSMS HPLC-UV	↓ High-quality grade I embryos ↓ Follicle size	-	-	32–35	79	[63]
↑ Alanine ↑ Lysine ↑ Phenylalanine ↑ Leucine ↑ Proline	NMR	-	-	I/II/III/IV	23–35	95/24	[122]
↑ Valine ↑ Fucose ↑ Choline-containing metabolites ↑ Glycerophosphocholine ↑ Lysine/arginine ↑ Lipoproteins ↓ Creatinine	1H- NMR	-	-	I/II/III/IV	25–37	50/23	[123]
↑ Lactate ↑ 3-Hydroxybutyrate ↑ Alanine ↑ Leucine ↑ Valine ↑ Threonine ↑ Lysine ↑ Glycerophosphatidylcholine ↑ Succinic Acid ↑ 2-Hydroxybutyrate ↓ Lipids ↓ Glucose ↓ Isoleucine ↓ Arginine	NMR		↑ Anaerobic glycolysis ↑ OS ↓ NO ↓ NOS	I/II	<40	22/22	[107]
↑ Homocysteine	Immunoassay	-	-	-	30–38	29/29	[50]
↓ Nesfatin-1	Immunoassay	-	↓ BMI	I/II/III/IV	21–35	25/25	[124]
↑ Glycodelin-A	Immunoassay	-	-	I/II/III/IV	26–49	58/40	[125]
↑ Glycodelin-A ↑ IL-6	Immunoassay	-	-	II/III/IV	21–48	48/20	[126]
↓ Haptoglobin	Immunoassay Biochemical assay	-	-	I/II/III/IV	27–40	15/15	[127]
↑ miR-515-5p ↑ miR-29b-1-5p ↓ miR-3168 ↑ miR-6502-5p ↑ miR-4748 ↓ miR-3137	Immunoassay	-	-	I/II/III/IV	20–42	100 /47	[128]
↓ let-7a-5p ↓ let-7b-5p ↓ let-7d-5p ↓ let-7f-5p ↓ let-7g- 5p ↓ let-7i-5p ↓ miR-199a-3p ↓ miR-320a ↓ miR-320b ↓ miR-320c ↓ miR-320d ↓ miR-328-3p ↓ miR-331-3p ↓ miR-320e	Immunoassay	-	-	-	-	29/10	[129]
↑ miR- 125b-5p ↑ miR-150-5p ↑ miR-342-3p ↑ miR-451a ↓ miR-3613-5p ↓ let-7b	Immunoassay	-	-	I/II/III/IV	27–41	41/59	[130]
↑ miR-22-3p ↑ miR-320a	Immunoassay	-	-	I/II/III/IV	20–50	20/20	[131]
↓ miR-155 ↓ miR574-3p ↓ miR139-3p	Immunoassay	-	-	I/II/III/IV	18–50	51/16	[132]
↑ miR-125b-5p ↑ miR-28-5p ↑ miR-29a-3p	Immunoassay	-	-	I/II/III/IV	27–36	82/38	[133]
↓ miR-30c-5p ↓ miR- 127-3p ↓ miR-99b-5p ↓ miRNA-15b-5p ↓ miRNA-20a-5p ↑ miR-424-3p ↑ miR-185-5p was	Immunoassay	-	-	I/II	21–43	30/20	[134]
↓ miR-3613-5p ↓ miR-6755-3p ↑ miR-125b-5p ↑ miR-150–5p ↑ miR-342-3p ↑ miR-143-3p ↑ miR-145-5p ↑ miR-500a-3p ↑ miR-451a ↑ miR-18a-5p	Immunoassay	-	-	III /IV	26–40	24/24	[135]
↓ miR-17-5p ↓ miR-20a ↓ miR-22	Immunoassay	-	-	III/IV	25–44	23/23	[136]
↑ miR-200c ↓ miR-34a-5p	Immunoassay	-	-	I/II/III/IV	-	71/65	[137]
↓ miR-31 ↑ miR-145	Immunoassay	-	-	-	-	-	[138]
↓ miRNA-154-5p ↓ miR-196b-5p ↓ miR-378a-3p ↑ miR-33a-5p	Immunoassay	-	-	III/IV	29–43	51/41	[139]
↓ let-7b ↓ miR-135a	Immunoassay	-	-	III/IV	26–40	24/24	[140]
↓ miR-200a ↓ miR-141	Immunoassay	-	-	I/II/III/IV	26–38	61/35	[141]
↑ miR-199a-5p	Immunoassay	-	-	-	-	33/65	[142]
↑ miR-9, 96 ↑ miR-182 ↑ miR-183 ↑ miR-196a ↑ miR-196b ↑ miR-205 ↑ miR-375	Immunoassay	-	-	-	-	33/20	[143]
↑ miR-199a ↑ miR-122 ↓ miR-145 ↓ miR-141 ↓ miR- 542-3p ↓ miR-9	Immunoassay	-	↑ Pelvic adhesion and distribution	I/II/III/IV	20–58	60/10	[144]
↑ CYP19A1 ↑ ESR1 ↑ ESR2 ↑ PGR ↑ BGN	Immunoassay	-	-	III/IV	26–34	11/9	[145]
↑ ITB3 ↑ ITA2B2 ↑ ACVL-1	Immunoassay	-	↑ Peritoneal Endometriosis	I/II	25–36	40/20	[146]
↑ CD48 ↑ DNAM-1 ↑ IL-31 ↑ XIAP	Immunoassay	-	↑ Apoptosis ↑ immune response	I/II/III/IV	23–40	68/35	[147]

## Data Availability

This systematic search was performed via the databases Web of Science, SCOPUS, b-on, and PubMed. The keywords and respective mergers used in the study were “follicular fluid” AND “metabolomics”, “metabolomics” AND “endometriosis”, “follicular fluid” AND “endometriosis”, “endometriosis” AND “follicular fluid” AND “metabolomics”, “serum” AND “metabolomics”, “serum” AND “endometriosis”, “endometriosis” AND “serum” AND “metabolomics”, “plasma” AND “metabolomics”, “plasma” AND “endometriosis”, and finally “endometriosis” AND “plasma” AND “metabolomics”. These had to be included in the article’s title, abstract, or key words. The research period ranged from 2010 to 2024. The studies considered were all human-related, with participants ranging in age, physical and metabolic characteristics, and ethnicity. Animal studies and studies that evaluated the response to any type of supplementation or extraordinary exposures were not included.
